# Targeting NK-1 Receptors to Prevent and Treat Pancreatic Cancer: A New Therapeutic Approach 

**DOI:** 10.3390/cancers7030832

**Published:** 2015-07-06

**Authors:** Miguel Muñoz, Rafael Coveñas

**Affiliations:** 1 Research Laboratory on Neuropeptides (IBIS), Virgen del Rocío University Hospital, 41013 Sevilla, Spain; 2 Laboratory of Neuroanatomy of the Peptidergic System (Lab. 14), Institute of Neurosciences of Castilla y León (INCYL), University of Salamanca, 37008 Salamanca, Spain; E-Mail: covenas@usal.es

**Keywords:** substance P, NK-1 receptor antagonist, chronic pancreatitis, inflammation, smoking, alcoholism, depression

## Abstract

Pancreatic cancer (PC) is the fourth leading cause of cancer related-deaths in both men and women, and the 1- and 5-year relative survival rates are 25% and 6%, respectively. It is known that smoking, alcoholism and psychological stress are risk factors that can promote PC and increase PC progression. To date, the prevention of PC is crucial because there is no curative treatment. After binding to the neurokinin-1 (NK-1) receptor (a receptor coupled to the stimulatory G-protein Gαs that activates adenylate cyclase), the peptide substance P (SP)—at high concentrations—is involved in many pathophysiological functions, such as depression, smoking, alcoholism, chronic inflammation and cancer. It is known that PC cells and samples express NK-1 receptors; that the NK-1 receptor is overexpressed in PC cells in comparison with non-tumor cells, and that nanomolar concentrations of SP induce PC cell proliferation. By contrast, NK-1 receptor antagonists exert antidepressive, anxiolytic and anti-inflammatory effects and anti-alcohol addiction. These antagonists also exert an antitumor action since *in vitro* they inhibit PC cell proliferation (PC cells death by apoptosis), and in a xenograft PC mouse model they exert both antitumor and anti-angiogenic actions. NK-1 receptor antagonists could be used for the treatment of PC and hence the NK-1 receptor could be a new promising therapeutic target in PC.

## 1. Introduction

Pancreatic cancer (PC), one of the most ominous types of cancer, is the fourth leading cause of cancer related-deaths in both men and women, with less than 5% survival at 5 years after diagnosis. In 2013, the American Cancer Society estimated that there were 45,220 new cases of PC in the United States and 38,460 deaths from the disease. Treatment strategies have not succeeded in significantly extending patient survival, and clinical outcome has not improved substantially over the past 35 years; the overall 5-year survival rate remains dismal, around 5% [1]. Thus, PC continues to be a major unsolved health problem and conventional treatments unfortunately have little impact on the course of the disease. Almost all patients suffering from PC develop metastases, this being the main reason for its lethality [2]. Cytostatic drugs show a low safety profile and severe side effects (e.g., anaemia, leukopenia), because these drugs are not specific against cancer cells. Currently, due to having the worst prognosis, a lack of early diagnostic symptoms, and resistance to conventional chemo- and radiotherapies, PC remains a very complex malignancy. Thus, because there is still a lack of curative therapy there is an urgent need to prevent PC by applying new strategies and/or by improving current therapies [3]. Research into this issue should focus on drugs with fewer side effects than those produced by cytostatic drugs and this can only be achieved if the drug is specific against PC cells.

**Figure 1:**
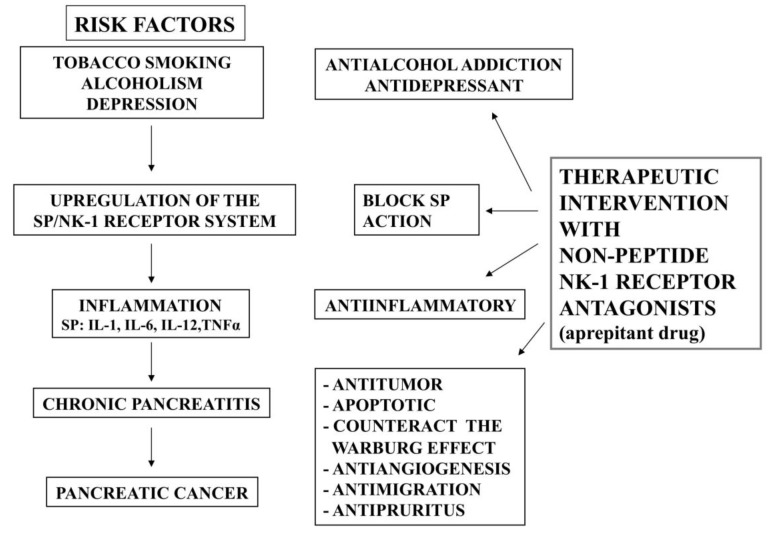
Risk factors (tobacco smoking, alcoholism, depression) for the development of PC are characterized by an up-regulation of the SP/NK-1 receptor system. This up-regulation is also present in inflammation and in chronic pancreatitis. Smoking and alcohol abuse induce a pro-inflammatory milieu and are risk factors for chronic pancreatitis; depression increases the level of SP, and chronic pancreatitis promotes PC. NK-1 receptor antagonists can prevent (by counteracting the risk factors/chronic pancreatitis) and can treat PC by blocking the pathophysiological actions exerted by SP via the NK-1 receptor.

In light of the above, a better understanding of both etiology and early developmental events in PC is vital. The evolution of advanced PC from initial pancreatic injury is a multi-factorial phenomenon involving a series of sequential events. The initial acute infection or tissue damage triggers inflammation, initiating the process of establishing a state of homeostasis in conjunction with innate immunity, aimed at limiting harm to the body. Upon recurrent pancreatic injuries, which may be due to genetic susceptibility, smoking and alcohol abuse, unhealthy diet, a pro-inflammatory milieu is induced; comprising several types of immune cells, growth factors, chemokines, cytokines and restructured extracellular matrix; this leads to prolonged inflammatory/chronic conditions [4]. Cells that have sustained DNA damage and/or mutagenic assault exploit the prolonged inflammatory response aiding in the initiation and development of neoplastic/fibrotic events. Many tumor-stromal interactions result in a chaotic environment, where loss of immune surveillance and repair response lead to PC [4]. Thus, the inflammatory process is crucial and a better understanding of the inflammatory markers defining this “injury-inflammation-cancer” pathway could aide in the identification of novel molecular targets for the treatment of PC. For example, during the inflammation process the substance P/neurokinin-1 receptor system is up-regulated and the neurokinin-1 receptor therefore is an important target for the treatment of inflammatory processes (Figure 1) [5].

It is known that the risk factor of PC is 13.6% for tobacco smoking and 13.0% for heavy alcohol drinking, and that the risk of PC increases to 25.7% when considering tobacco smoking and alcohol together [6]. This means that an appreciable proportion of the PC population could be avoided by modifying lifestyle habits such as tobacco smoking and heavy alcohol drinking. Smoking and alcoholism are well-established risk factors of chronic pancreatitis and PC [6] and chronic pancreatitis is a risk factor for the development of PC [7] (Figure 1). Moreover, it has been reported that chronic stress increases susceptibility for developing pancreatitis and accelerates PC growth and invasion [8,9].

Peptides are widely involved in human pathology (see [5] for a review). As indicated above, one of these peptides is the undecapeptide substance P (SP). The biological action of SP is mainly mediated by the tachykinin neurokinin-1 receptor (NK-1 receptor), since SP shows the highest affinity for the NK-1 receptor [10]. Many data indicate that the SP/NK-1 receptor system is involved in smoking, alcoholism, depression, chronic inflammation and cancer progression (Figure 1) [5,11,12]. This is very important, since it means that a therapeutic intervention with NK-1 receptor antagonists by targeting NK-1 receptors could improve the above pathologies (Figure 1). This strategy opens up new approaches for translational research. In view of this, the aim of this paper is to review the involvement of the SP/NK-1 receptor system in these pathologies and, in particular, in PC.

## 2. Substance P and the NK-1 Receptor 

The tachykinin family of peptides includes peptides such as SP, neurokinin A, neurokinin B, ranakinin, kassinin, neuropeptide gamma and eledoisin. SP is derived from the preprotachykinin-A gene and it acts as a neurotransmitter or neuromodulator in the nervous system. SP is ubiquitous throughout the body and the organic fluids (e.g., blood, cerebrospinal fluid, breast milk) [5].

Three receptors, designated NK-1, NK-2 and NK-3, are the mediators of the biological activities of tachykinins [10]. The NK-1 receptor has been identified as a regulator of the biological functions encoded by the C-terminal sequence of tachykinins [13]. SP shows the highest affinity for the NK-1 receptor and it is the natural ligand of this tachykinin receptor. After binding to the NK-1 receptor, SP acts in a concentration-dependent manner regulating many biological functions (e.g., emotional stress, neurogenic inflammation, alcohol addiction, mitogenesis, angiogenesis, emesis, pain, chemotaxis of leukocytes, pruritus) [5,10,14]. SP stimulates mitogenesis in cancer cells as it activates members of the mitogen-activated protein kinase (MAPK) family, which includes p38MAPK and the extracellular signal-regulated kinases 1 and 2 (ERK1/2) [14,15]. Upon activation, ERK1/2 is translocated into the nucleus where it induces proliferation and protects the cell from apoptosis [16]. Moreover, SP increases the intracellular calcium levels, this being associated with mitogenesis [17]. The NK-1 receptor is a G protein-coupled receptor (GPCR) that transmits the SP signal. Upon stimulation by SP, the NK-1 receptor interacts with multiple G proteins, including Gαs, Gαq/11, Gαi/o, Gα12 and Gα13 [18]. SP/NK-1 receptor binding can generate second messengers that trigger numerous effector mechanisms involved in cellular excitability and in the regulation of cellular function. This may include arachidonic acid mobilization via phospholipase A2; cAMP accumulation via stimulation of adenylate cyclase; stimulation, via phospholipase C, of phosphatidyl inositol turnover, leading to calcium mobilization [10]. It is also known that SP produces glycogen breakdown and that the glucose obtained is used by cells to increase their metabolism (glycolysis) [19]. After SP binds to the NK-1 receptor, both are internalized into endosomes. SP induces a clathrin-dependent internalization of the receptor; then SP is degraded and the receptor is recycled to the cell surface [10].

In cancer cells, the NK-1 receptor is mainly located in both plasma membrane and cytoplasm, although occasionally it is also observed in the nucleus of these cells [14]. The involvement of this receptor in acute pancreatitis through mediation of neurogenic inflammation has been reported [18]. In humans, two subtypes of the NK-1 receptor have been described: the truncated and full-length forms. The former enhances the growth of cancer cells and stimulates the production of cytokines, which up-regulate the truncated form, whereas the second mediates a slow growth of cancer cells [20]. Cytokines activate the transcription factor called NF-κB, which in turn up-regulates the truncated subtype but only slightly increases the full-length subtype [18,21,22]. In tumor cells, the truncated form mediates malignancy and is more abundant in colonic epithelial cells from patients with colitis-associated cancer. However, the full-length form is not affected [20,23].

## 3. Smoking, Pancreatitis and the SP/NK-1 Receptor System 

Cigarette smoke is a complex mixture of nicotine and carcinogenic compounds, many of which have deleterious effects on the exocrine pancreas. In fact, nicotine exerts toxic effects on the pancreas, forms carcinogenic N-nitroso compounds during smoking [24,25,26] and, in the exocrine pancreas, it induces cytoplasmic vacuolization, cellular oedema and increases cellular amylase content [27]. Nicotine inhibits the secretion of bicarbonate and affects the composition of pancreatic secretion and, in patients suffering pancreatitis, nicotine exposure results in increased pancreatic enzyme secretion, including amylase and lipase [28,29,30,31]. 

It has been reported that exposure of rats to tobacco smoke produced fibrosis and scarring of pancreatic acinar structures, characteristic marks of chronic pancreatitis [32]. In this sense, it is also known that the SP/NK-1 receptor system is up-regulated during the inflammation processes (Figure 1) and that, in rats, NK-1 receptor antagonists exert an anti-inflammatory action [5,13]. SP is a key mediator in neurogenic inflammation [33], the capsaicin-sensitive primary afferent neurons being responsible for this type of inflammation in peripheral organs [34]. In acute pancreatitis, the SP/NK-1 receptor system plays an important role through the mediation of neurogenic inflammation [18]. The hallmarks of neurogenic inflammation are an increase in vascular permeability, plasma extravasation, oedema formation, and leukocyte infiltration [33,34]. In addition, in inflammatory processes, SP contributes to pain transmission [34]. The NK-1 receptor is present in the immune system [18]. SP is also an immunomodulator that, in particular, regulates the immune function of mononuclear phagocytes. SP activates NF-κB [35], a transcription factor involved in the control of the expression of inflammatory cytokines, and the peptide also stimulates human peripheral blood monocytes to produce inflammatory cytokines, including IL-1, IL-6, IL-12, and TNFα [36]. Moreover, it is known that NK-1 receptor antagonists decrease pro-inflammatory signals [18].

Cigarette smoke stimulates macrophages to synergize lipopolysaccharide (LPS)-evoked IL-1β and TNFα secretion and amplifies the LPS-induced macrophage secretion of SP, playing an important role in generating the cytokines’ synergy by acting on the NK-1 receptor [12]. It is known that NF-κB up-regulates the truncated NK-1 receptor; that NF-κB activation is essential for the genesis of this cytokines’ synergy, and that both the NK-1 receptor and the phosphatidyl-3-kinase (PI3K)/Akt pathways are responsible for the NF-κB response to cigarette smoke condensate plus LPS [12]. This could explain the phenomenon by which LPS induces a much greater secretion of IL-1β and TNFα from alveolar macrophages in smokers than in healthy non-smokers [12]. It has been also demonstrated that after blockade of NK-1 receptors, the responses of IL-1β, TNFα and NF-κB activation are reduced, and that NF-κB inhibitors decrease the response of the cytokines [12]. Thus, by means of these strategies the formation/development of a pro-inflammatory milieu could be counteracted. 

For the development of PC, epidemiological studies have concluded that tobacco smoking is the highest risk factor (Figure 1) [37,38,39,40]. This risk increases, depending of the quantity of tobacco consumed and of the number of years of smoking. Although smokers who are able to quit smoking can reduce their risk of developing PC by nearly 50% within two years of quitting, the risk still remains higher than that of non-smokers for 10 years. [37]. It is also known that cigarette smoking accelerates the progression of alcoholic chronic pancreatitis [41].

## 4. Alcoholism, Pancreatitis and the SP/NK-1 Receptor System 

Alcohol is the factor most frequently associated with chronic pancreatitis and the risk of PC is very high in subjects suffering from chronic pancreatitis [7,40] (Figure 1). In fact, the relative risk of chronic pancreatitis would be multiplied approximately by a factor of 1.4 per 20 g increase in alcoholic intake [42]. It has been suggested that NK-1 receptor antagonists may be promising new drugs for the treatment of alcohol addiction [11]. The role played by alcohol is the cornerstone of the pathogenesis of alcoholic chronic pancreatitis [43]. The pancreatic functional changes caused by alcoholic pancreatitis progress even after the cessation of alcohol use, but the progression is slower and less severe when alcohol intake is stopped [44]. The possible mechanisms by which alcohol sensitizes the pancreas, inducing a chronic injury, have been reported [45]. Rats treated with ethanol (for 8 weeks) and cyclosporin A (for the last two weeks), and in which in addition an acute pancreatitis was induced by cerulein, a massive loss of acinar cells, persistent inflammatory infiltration and fibrosis were observed in comparison to that found in control animals receiving cyclosporin A plus cerulein. In ethanol-treated rats, macrophages were prominent in the inflammatory infiltrate and the animals showed a marked increase in pancreatic NF-κB activation, in cytokine/chemokine mRNA expression, in collagen and fibronectin, in the expression and activities of matrix metalloproteinases 2 and 9, and in the activation of pancreatic stellate cells [45]. 

In addition, protein 43, which is overexpressed in alcoholic chronic pancreatitis, stimulates the synthesis of SP, and the activation of peripheral nerve fibers also induces the synthesis/release of SP [43,46]. Through the NK-1 receptor, SP stimulates inflammatory cells to produce cytokines (IL-1, IL-6, IL-12, TNFα) and it increases the number of poly-morphonuclear cells, macrophages and fibroblasts. After neurogenic inflammation, it seems that the components of the visceral pain pathways are sensitized [47]. In chronic pancreatitis, pain and neurogenic inflammation could be related, being presented as a possible amplifier of the noxious signal from the pancreas. It has been reported that in chronic pancreatitis patients, with a mild to moderate and strong intensity of pain, that NK-1 receptor mRNA levels increase 14- and 30-fold over controls, respectively. In the same patients, a significant relationship between NK-1 receptor mRNA levels and the intensity of pain, between NK-1 receptor mRNA levels and the frequency of pain, and between NK-1 receptor mRNA levels and the duration of pain has been reported, but not regarding the degree of tissue inflammation [48]. Thus, NK-1 receptor signaling may play a role in the pain syndrome of chronic pancreatitis. The expression of NK-1 receptors in blood vessels and in inflammatory cells also suggests a possible interaction of inflammatory cells, blood vessels and immunoreactive SP nerves, further supporting the existence of a neuroimmune interaction that probably influences pain syndrome and chronic inflammatory changes characteristic in chronic pancreatitis [48].

## 5. Depression, Pancreatic Cancer and the SP/NK-1 Receptor System 

Cancer and depression commonly occur at the same time. The prevalence of depression among cancer patients often increases with disease severity and upon the appearance of symptoms such as pain and fatigue. In the same person, cancer and depression occur very frequently, and in fact the development of cancer is more likely in patients suffering from severe or chronic depression. In cancer patients, the prevalence of depression increases with the severity of the disease. Many data support the idea that depression predicts cancer progression and mortality and that psychosocial support increases the survival time of patients with cancer by reducing anxiety and depression. Thus, a bi-directional relationship between depression and cancer must occur, and hence new strategies are now emerging for adequate therapeutic intervention [49]. In this sense, it has been reported that mammary tumorgenesis is related to the exposure of several forms of stressors together with factors associated with life-style. In breast cancer, a crucial factor in tumor progression is the interaction among the following items: psychosocial support, personality and stress, as well as the result of this interaction on an individual's ability to cope with stress [49,50]. In sum, all these data support the idea that psychological factors play an important role in cancer progression [50]. 

In several regions of the CNS the expression of NK-1 receptors and that of the genes encoding the synthesis of tachykinins are modified after the administration of psychotropic drugs [51,52]. Thus, in the CNS (e.g., substantia nigra, amygdala, striatum), chronically administered antidepressant drugs decrease the concentration of SP in these regions. This means that by decreasing the level of SP in several central nervous regions, antidepressant drugs could exert a beneficial effect in the treatment of affective disorders [53].

It is known that both SP and NK-1 receptors are widely distributed in the limbic system (amygdala, hypothalamus) and that SP plays an important role in the integration of emotional responses to stress, suggesting that an alteration in the SP/NK-1 receptor system is responsible for the pathogenesis of depression (Figure 1). In fact, an increase in the level of SP has been reported in this disease [54] and NK-1 receptor antagonists (L-733,060, the drug aprepitant) have been used as antidepressive agents [54]. Latter, in other study, aprepitant (160 mg) was administered to patients suffering a major depressive disorder and the authors found a lack of efficacy of the drug [55]. Thus, the use of aprepitant as an antidepressive drug is debatable [54,55]. However, there is an important point to note; the doses of aprepitant used in both studies: 300 mg/day [54] and 160 mg/day [55]. It seems that the different doses used could be responsible for the contradictory results. Despite initial findings in support of the antidepressant activity of NK-1 receptor antagonists in humans, the clinical efficacy of these indications has not been appropriately checked. Further studies must be carried out in order to check the possible antidepressive action of aprepitant. 

It has been reported that both aprepitant and L-733,060 have antitumor activity against many and different human cancer cell lines, including PC cells [3,56,57]. The data suggest that in depression the SP/NK-1 receptor system could be activated (Figure 1) and hence the disease could facilitate the proliferation of PC cells, since these cells overexpress the NK-1 receptor. Thus, after binding to NK-1 receptors located in the neurons of the limbic system SP induces depression [54], and after binding to NK-1 receptors expressed in PC cells the same peptide elicits tumor cell proliferation and an antiapoptotic effect. This means that there is a neuroendocrine link and cross talk between the central nervous system and the tumor mass, because in both depression and PC SP is overexpressed and released into the blood and hence SP plasma levels increase. In sum, many data suggest that emotional behaviour (e.g., depression, anxiety) [58,59] and cancer progression [60] could be related because an alteration occurs in the SP/NK-1 receptor system (Figure 1). NK-1 receptor antagonists exert antitumor and antidepressant actions and for this reason it should be very important to treat depression with NK-1 receptor antagonists because they improve the symptoms of depression and could prevent/improve PC. 

## 6. Chronic Pancreatitis and the SP/NK-1 Receptor System

It has been well established that the SP/NK-1 receptor system plays a crucial role in chronic inflammatory diseases (e.g., chronic pancreatitis, ulcerative colitis, bowel disease) [5,10,61]. As indicated above, the risk of PC is very high in subjects suffering from chronic pancreatitis and appears to be independent of sex, country, or type of pancreatitis [7,40]. Inflammation increases both mitogenesis and mutagenesis [62]. A dividing cell is known to be at greater risk of mutation than is a quiescent one, and cell division allows adducts to convert to mutations [63]. The time interval for DNA repair during cell division is short, and hence the risk of endogenous or exogenous damage is higher if cells are proliferating. Inflammation may become chronic either because an inflammatory stimulus persists or because of deregulations in the control mechanisms that would normally terminate the process. Many of the cells, cytokines and processes (e.g., leukocyte migration, angiogenesis) involved in the inflammation are also observed in tumors. It is known that tachykinins control the activity of inflammatory cells; that SP induces oedema formation, and that both SP and NK-1 receptors are up-regulated during inflammation processes [5] (Figure 1). Taking together, the data suggest that chronic inflammation could facilitate the development of PC through the SP/NK-1 receptor system because this system is up-regulated in inflammatory processes and it is known that SP elicits PC cell proliferation and that PC cells overexpress the NK-1 receptor [3,56,64,65]. In addition, the up-regulation of NK-1 receptor mRNA expression in chronic pancreatitis is tightly related to the pain syndrome experienced by these patients [48]. 

Overall, the data support the idea that a new strategy in the prevention of PC may be possible, namely a prophylactic therapy using NK-1 receptor antagonists in chronic pancreatitis to prevent PC, because these antagonists exert an anti-inflammatory and an anti-proliferative effect. Accordingly, the treatment with NK-1 receptor antagonists could prevent the appearance/progression of PC [3,14]. 

## 7. Pancreatic Cancer and the SP/NK-1 Receptor System 

It has been shown that SP controls important biological functions related to cancer, such as tumor cell proliferation, migration of tumor cells for invasion, infiltration and metastasis, neoangiogenesis, and it exhibits an antiapoptotic effect on cancer cells [3,5,10]. SP increases phosphorylation and activity of Akt or protein kinase B, a serine protein kinase that becomes activated via phosphatidyl-3-kinase (PI3K) in cancer cells, which in turn suppresses apoptosis [66,67]. SP induces the release of glutamate, taurine and interleukins from cancer cells and as a result induces an inflammatory process that increases the levels of SP and hence increases cancer cell proliferation [68,69,70]. At nanomolar concentrations, SP elicits cancer cell proliferation [3,10,14] and exerts an antiapoptotic effect [16]. PC cells and samples express NK-1 receptors and it has been observed that human PC cell lines express more NK-1 receptors than non-tumor cells (overexpression). In pancreatic samples from patients with advanced tumor stages there are significantly higher levels of the NK-1 receptor, where higher rates of NK-1 receptor expression are associated with advanced tumor stages and a poorer prognosis [10,14,64]. This data suggest a correlation between the prevalence of NK-1 receptors in cells with the degree of malignancy. Moreover, the overexpression of the NK-1 receptor in PC cells suggests the possibility of using specific drugs (e.g., NK-1 receptor antagonists) against PC cells. Thus, decreasing the side effects observed when non-specific drugs (cytostatics) are used.

In cancer cells, glucose is produced when SP stimulates glycogen breakdown [19] by glycolysis producing energy that is followed by lactic acid fermentation (this is called the Warburg effect). Cancer cells exhibit glycolytic rates up to 200 times higher than those observed in normal tissue of origin. Thus, SP released from cancer cells produces glycogen breakdown and the glucose obtained is used by cancer cells to increase their metabolism (glycolysis) [19]. This process is blocked by NK-1 receptor antagonists and then, in tumor cells, glycolysis is not possible when glucose is absent.

By western blot, it has been demonstrated that human CAPAN-1 and PA-TU 8902 PC cell lines express several glycosylated isoforms (34, 46, 58 and 75 kDa) of the NK-1 receptor [56]. Until now the possible physiological implications of this finding remain unknown, although it has been suggested that the glycosylation of the NK-1 receptor may stabilize the receptor in the plasma membrane [71]. Moreover, the up-regulation of the NK-1 receptor is crucial for both NK-1 receptor function and cancer proliferation [14], since the NK-1 receptor is not functional when it is down-regulated [16]. It is also known that the NK-1 receptor is involved in the viability of the tumor cells, since following the application of a knockdown method (small interfering RNA gene-silencing (siRNA)), tumor cells die by apoptosis [14]: more apoptotic cells were observed in siRNA cells than in non- transfected cells and hence the number of siRNA tumor cells was significantly lower. Thus, it seems that in cancer cells glycosylation of the NK-1 receptor is another pathway for the activation of these cells, in addition to the overexpression of NK-1 receptors. 

The migration of cancer cells is a crucial requisite for the development of metastasis and cancer progression. Over 90% of the cancer related-deaths are derived not from the primary tumor but from metastases [72]. Thus, a major goal in the treatment of cancer should be to inhibit the spread of cancer cells to other tissues. Tumor cell migration is induced by peptides (e.g., SP) and classical neurotransmitters (dopamine, noradrenaline); this migration is inhibited after D_2_ receptor, adrenoceptor or NK-1 receptor antagonists [10,60] are administered. SP induces a rapid change in cellular shape (including blebbing) after binding to the NK-1 receptor. Membrane blebbing is important in cell spreading, cancer cell infiltration, and cell movement [73,74].

SP is involved in PC perineural invasion and in PC cells facilitates invasion and proliferation and the expression of matrix metalloproteinase (MMP)-2 [75]. Moreover, SP induces neurite outgrowth and the migration of PC cell clusters to the dorsal root ganglia of newborns [75]. By contrast, NK-1 receptor antagonists exert an antitumor action against PC *in vitro* and *in vivo* [56]. Thus, NK-1 receptor antagonists (e.g., L-733,060, aprepitant) elicit antitumor activity against CAPAN-1 and PA-TU 8902 PC cell lines in a concentration dependent manner [56,57]. This action occurs because after binding to the NK-1 receptors located in pancreatic cells, NK-1 receptor antagonists induce apoptosis in the tumor cells. NK-1 receptor antagonists exert a dual action on PC: they inhibit both PC cell proliferation and angiogenesis [76], since it is also known that SP facilitates angiogenesis [14]. SP facilitates the proliferation of endothelial cells, stimulating vessel growth and increasing tumoral blood flow, both of which are crucial for tumor development [77,78]. However, NK-2 and NK-3 agonists do not exert significant effects on the proliferation of endothelial cells. Early neoangiogenesis is a key step in the transition from acute to persistent inflammation. In fact, SP and the NK-1 receptor have been observed in intra- and peri-tumoral blood vessels, and during neoangiogenesis both the expression of NK-1 receptors and tissue innervation are increased [78,79]. NK-1 receptor antagonists attenuated significantly the growth of HPAF-II tumor xenografts in nude mice, reduced tumor-associated angiogenesis and inhibited Ca^2+^ mobilization and DNA synthesis in HPAF-II PC cell line [76].

In sum, to date the data indicate that the administration of NK-1 receptor antagonists (Figure 1) is an excellent tool for the treatment of chronic pancreatitis induced by smoking and alcoholism, for the treatment of depression-cancer development, and for PC. This means that the NK-1 receptor is an important target for the treatment of these pathologies.

## 8. NK-1 Receptor Antagonists for the Prevention and Treatment of Pancreatic Cancer 

NK-1 receptors antagonists form a broad group of heterogeneous compounds with distinct chemical compositions and the same stereochemical features. The pharmacologic effect of NK-1 receptor antagonists (acting in a concentration-dependent manner) is related to stereochemical features and it is not linked to the chemical composition. There are two groups of NK-1 receptor antagonists: peptide and non-peptide. The former (e.g., Spantide I and II, SP (4–11), NY-3,238; NY-3,460) are subject to a number of drawbacks: poor potency; partial residual agonist activity; the inability to discriminate between tachykinin receptors; neurotoxicity, and mast cell degranulating activity [13]. *In vitro* and *in vivo*, the antagonist [d-Arg^1^, d-Trp^5,7,9^, Leu^11^] SP has shown antitumor effects (e.g., in PC) [76,80,81,82,83].

For non-peptide NK-1 receptor antagonists and SP the binding sites are different [84]. Whereas SP (hydrophilic) binds to the extracellular ends of the transmembrane helices, and especially to the extracellular loops of the receptor, the antagonists (small molecules and lipophilic) bind more deeply between the transmembrane III-VII domains. For example, non-peptide NK-1 receptor antagonists include the following compounds: perhydroisoindolones (RP-67,580, RP-73,467, RPR-100,893), steroids (WIN-51,708), tryptophan based (L-732,138, L-737,488), benzylamino and benzyl ether quinuclidines (L-709,210, CP-96,345), benzyl ether piperidines (L-733,060, L-741,671, L-742,694), benzylamino piperidines (CP-99,994, GR-203,040, GR-205,171, CP-122,721) [13]. Some of these non-peptide NK-1 antagonists have been used in clinical trials and found to be safe; this is the case for the drug aprepitant and its prodrug fosaprepitant, casopitant (GW-679,769), vofopitant (GR-205,171), L-759,274, CP-122,721, ezlopitant (CJ-11,974), rolapitant, L-754,030, serlopitant and CJ-11,974 [84]. Non-peptide NK-1 receptor antagonists exert the following pharmacological effects: antidepressant, anxiolytic, anti-inflammatory, anti-alcohol addiction, antiemetic, antimigraine, neuroprotector, analgesic, hepatoprotector, antivirus proliferation [5]. However, aprepitant (Emend, MK-869, L-754,030) and its intravenously administered prodrug fosaprepitant (Ivemend, MK-0517, L-758,298) are the only non-peptide NK-1 receptor antagonists currently used in clinical practice (for the treatment of acute and delayed chemotherapy-induced nausea and vomiting and post-operative nausea and vomiting) [85]. Chemotherapy induces the release of SP and aprepitant blocks the unwanted actions exerted by SP [86]. The safety of aprepitant (e.g., 300 mg/day is well tolerated) has been confirmed in many human clinical trials [85] and in human fibroblasts, in which the IC_50_ is three times higher than the IC_50_ for cancer cells [57]. Moreover, it is known that the IC_50_ for non-tumor cells is 90 μM but the IC_100_ for tumor cells is approximately 60 μM [57]. 

Non-peptide NK-1 receptor antagonists could be considered a new generation of antitumor broad-spectrum drugs [5,10], since these antagonists (L-733,060, L-732,138, aprepitant) exert an antitumor action, inducing tumor cell death by apoptosis, including PC cells. Furthermore, these receptor antagonists inhibit the migration of tumor cells and have anti-angiogenesis effects [3,10,14,57]. For example, the antitumor action of aprepitant against PC cells has been reported: this non-peptide NK-1 receptor antagonist inhibits (PC cells die by apoptosis) 100% of PC cells in a concentration-dependent manner [57].

**Figure 2:**
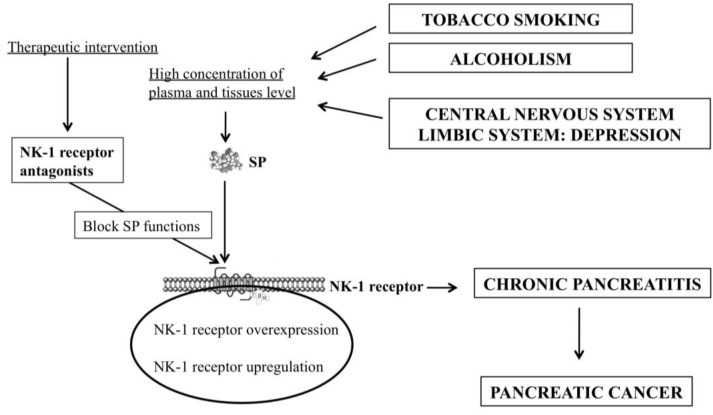
Tobacco smoking, alcoholism and depression increase the level of SP. NK-1 receptor antagonists block the pathophysiological actions mediated by SP. Chronic pancreatitis could facilitate the development of PC through the SP/NK-1 receptor system because this system is up-regulated in inflammatory processes, SP elicits PC cell proliferation and PC cells overexpress the NK-1 receptor.

In sum, according to the therapeutic features of non-peptide NK-1 receptor antagonists and to their pharmacological profile, the use of these compounds could prevent PC because counteract the main risk factors of PC (smoking, alcoholism, depression) and they could be also used for the treatment of chronic inflammation/PC (Figure 1 and Figure 2). It is important to note that smoking and alcoholism are involved in the inflammation of the pancreas and that chronic inflammation is a risk factor for developing PC, that is independent from the origin of the chronic pancreatitis. In both chronic pancreatitis and PC there is an overexpression of the NK-1 receptor and this receptor is also involved in smoking, alcoholism and depression. That is, the five pathologies are linked by the NK-1 receptor and this indicates that the SP/NK-1 receptor system plays an important role in these pathologies. This suggests that non-peptide NK-1 receptor antagonists (e.g., aprepitant) could be used for the prevention/treatment of PC and hence the NK-1 receptor could be a new promising therapeutic target in PC (Figure 1 and Figure 2).

Pruritus can occur as a paraneoplastic sign in cancer. Pruritus is a nociceptive stimulus mediated by SP that has a high impact on the quality of life of patients. It has been reported that patients with chronic pruritus and treated with the NK-1 receptor antagonist aprepitant underwent a considerable reduction in itch intensity [87]. These results are promising enough to warrant studies aimed at confirming the efficacy of NK-1 receptor antagonists in the treatment of pruritus.

## 9. Conclusions

The SP/NK-1 receptor system is up-regulated in smoking and alcoholism which are risk factors for developing chronic pancreatitis, in depression, in chronic pancreatitis and in PC (Figure 1 and Figure 2). In chronic pancreatitis and in PC, an overexpression of the NK-1 receptor occurs. This overexpression suggests the possibility of a specific treatment against PC cells using NK-1 receptor antagonists and a considerable decrease in the side effects produced, in comparison with those observed when cytostatics drugs are administered. SP is also increased in depression and in inflammatory processes. After binding to the NK-1 receptor, SP exerts a mitogenic/antiapoptotic action in PC cells. SP also regulates the migration of tumor cells, angiogenesis and the Warburg effect. This peptide could induce mitogenesis and PC progression via the following mechanisms: paracrine (SP is secreted by non-tumor cells, e.g., inflammatory cells; SP stimulates the mitogenesis in the endothelial cells favouring neoangiogenesis); SP is released from nerve terminals; endocrine (SP is released from the PC tumor mass into the blood vessels); SP reaches the whole body through the bloodstream (this is regulated by the limbic system). Currently, the presence of SP in PC cells has not been reported. However, an autocrine action should not be discarded, since many human cancer cell lines synthesize/release SP.

The truncated NK-1 receptor appears to be oncogenic and its expression is controlled by NF-κB. PC cells need the NK-1 receptor for their own survival, because they depend on the potent mitotic signal mediated by SP, and it seems that by means of the overexpression of the NK-1 receptor PC cells neutralize their own pathways, leading to cell death. SP also protects PC cells from apoptosis. By contrast, the absence of the mitotic signal mediated by SP when the NK-1 receptor is blocked with non-peptide NK-1 receptor antagonists could tilt the balance within the cell, favouring apoptotic/death signals and hence cell death. The NK-1 receptor can thus be considered a target in PC treatment and prevention.

NK-1 receptor antagonists inhibit the proliferation (inducing the apoptosis of PC cells) and the migratory activity of tumor cells; they exert an anti-angiogenic effect, and they counteract the Warburg effect. In addition, these antagonists could exert anti-inflammatory and antidepressive effects and anti-alcohol addiction (Figure 1). In general, non-peptide NK-1 receptor antagonists are safe and well tolerated in humans. To date, more than 300 non-peptide NK-1 receptor antagonists have been reported and the question therefore arises as to which is the best non-peptide NK-1 receptor antagonist for use in the prevention/treatment of chronic pancreatitis and PC. The answer is aprepitant, because this drug is used in clinical practice and exerts antitumor action against a large number of human cancer cell lines, including PC cells. Accordingly, many of the required safety and characterization studies for aprepitant have already been carried out. In a near future, the antitumor/antimigratory action of aprepitant against PC and the anti-angiogenic action of this drug should be fully tested in human clinical trials. It seems that by increasing the number of days on which aprepitant is currently administrated in clinical practice (three days), and using higher doses than those used for chemotherapy-induced nausea and vomiting, this non-peptide NK-1 receptor antagonist could be effective in the prevention/treatment of chronic pancreatitis and PC.
